# TNEA therapy promotes the autophagic degradation of NLRP3 inflammasome in a transgenic mouse model of Alzheimer’s disease via TFEB/TFE3 activation

**DOI:** 10.1186/s12974-023-02698-w

**Published:** 2023-02-02

**Authors:** Wenjia Lin, Zhao Li, Guangfeng Liang, Runjin Zhou, Xiaoyan Zheng, Rongrong Tao, Qingwei Huo, Chengfu Su, Min Li, Nenggui Xu, Chunzhi Tang, Ju-Xian Song

**Affiliations:** 1 Clinical Medical College of Acupuncture-Moxibustion and Rehabilitation, Guangzhou University of Chinese Medicine, Guangzhou, China; 2 Department of Acupuncture and Moxibustion, The Affiliated TCM Hospital of Guangzhou Medical University, Guangzhou, China; 3 School of Rehabilitation Sciences, Southern Medical University, Guangzhou, China; 4 Mr. & Mrs. Ko Chi-Ming Centre for Parkinson’s Disease Research, School of Chinese Medicine, Hong Kong Baptist University, Hong Kong SAR, China

**Keywords:** Alzheimer’s disease, NLRP3 inflammasome, Autophagy, Transcription factor EB, Electroacupuncture

## Abstract

**Background:**

The impairment in the autophagy-lysosomal pathway (ALP) and the activation of NLR family pyrin domain containing 3 (NLRP3) inflammasome represent two molecular events leading to neurodegeneration and neuroinflammation in Alzheimer’s disease (AD), a devastating neurodegenerative disorder without a cure. Previously we demonstrated the cognitive-enhancing effect of a combined electroacupuncture (EA) therapy termed TNEA in a transgenic mouse model of AD, involving activation of transcription factor EB (TFEB), a master regulator of ALP. However, whether and how TNEA inhibits NLRP3 inflammasome via TFEB-mediated ALP in AD remains to be investigated.

**Methods:**

5xFAD mice overexpressing amyloid-β (Aβ) were treated with TNEA or EA on its composing acupoints (GB13 and GV24). The changes in the signaling pathways regulating NLRP3 inflammasome, the association of NLRP3 inflammasome with ALP, and the roles of TFEB/TFE3 in mice brains were determined by immunoblots, immunohistochemistry and AAV-mediated knockdown assays.

**Results:**

TNEA inhibits the activation of NLRP3 inflammasome and the release of active interleukin 1β (IL1B) in the hippocampi of 5xFAD mice. Mechanistically, TNEA promoted the autophagic degradation of inflammasome components via activating both TFEB and TFE3 by modulating kinases including AMPK and AKT. The composing acupoints in TNEA showed synergistic effects on regulating these molecular events and memory improvement.

**Conclusion:**

Our findings suggest that TNEA attenuates AD-associated memory impairment via promoting TFEB/TFE3-mediated autophagic clearance of Aβ and NLRP3 inflammasome, and partially reveal the molecular basis of combined acupoints therapy originated from ancient wisdom.

**Supplementary Information:**

The online version contains supplementary material available at 10.1186/s12974-023-02698-w.

## Background

Alzheimer’s disease (AD), the most prevalent and devastating neurodegenerative disorder (ND), has been increasingly considered as an inflammasomopathy in which neuroinflammation plays a central role in its pathogenic development [1, 2]. The NLR family pyrin domain containing 3 (NLRP3) inflammasome, a protein complex formed by NLRP3 and the adaptor proteins, apoptosis-associated speck-like protein containing a CARD (ASC, also termed as PYCARD) and caspase 1 (CASP1), can be activated by β-amyloid (Aβ) and microtubule-associated protein tau (MAPT) proteins, two most acknowledged pathological hallmarks of AD, followed by cleavage and activation of cytokines such as interleukin-1β (IL1B) and IL18 leading to neuronal dysfunction and pyroptosis [3–7]. Accordingly, NLRP3 inflammasome inhibitors have been shown to attenuate Aβ pathology and rescue cognitive impairment in animal models of AD [8–11].

The dysfunction of the autophagy-lysosomal pathway (ALP), a cellular process responsible for degradation of protein aggregates, damages organelles and invading pathogens via lysosomes, also plays key roles in the pathogenesis of NDs including AD [12–16]. Among multiple signaling pathways regulating ALP, transcription factor EB (TFEB) and transcription factor binding to IGHM enhancer 3 (TFE3) have been identified as master regulators [17–19]. We and others have demonstrated the roles of deficient TFEB/TFE3-mediated ALP in the pathogenesis of AD [20, 21] and the potential application of TFEB activators for treating AD [22–24]. Importantly, ALP interplays with NLRP3 inflammasome. ALP inhibits NLRP3 inflammasome by promoting the autophagic degradation of inflammasome triggers, inflammasome components and cytokines [25]. Therefore, targeting ALP may represent a more efficient strategy to limit NLRP3 inflammasome in NDs [26]. For example, a natural compound thonningianin A promoted the autophagic degradation of the Aβ and NLRP3 inflammasome, thus improving cognitive function in APP/PS1 mice via inducing AMP-activated protein kinase (AMPK)/Unc-51-like kinase 1 (ULK1)-mediated autophagy [27]. However, whether activation of TFEB/TFE3 promotes the autophagic degradation of NLRP3 inflammasome and cytokines, thus improving memory in animal models of AD has not been reported.

We have previously demonstrated the neuroprotective effects of a combined electroacupuncture (EA) therapy termed TNEA in the 5xFAD transgenic mouse model of AD, and revealed the molecular mechanisms involving TFEB-mediated autophagic degradation of Aβ [28]. In this study, we addressed whether TNEA inhibits the activation of NLRP3 inflammasome and release of proinflammatory cytokines in AD mouse brain via enhancing TFEB/TFE3-mediated ALP, and unveiled the relevant molecular basis of acupoints combination in TNEA.

## Methods

### Study design

This study aimed to determine the anti-inflammatory effect of TNEA mediated by autophagic degradation of NLRP3 inflammasome in 5xFAD mice. The sample size (*n* = 5–11) in each experiment was determined based on experience from our previous study. 5xFAD mice were randomly allocated into the control and the intervention groups, with C57/BL6J mice as wild-type controls. The investigators who perform the statistical analysis were blind to the grouping.

### Reagents and antibodies

Anti-phospho TFEB (Ser142) (ABE1971) and anti-TFE3 (HPA023881) were purchased from Sigma-Aldrich. Anti-H3F3A/histone H3 (D1H2; 4499), anti-phospho-AKT (S473), anti-AKT (9272), anti-AMPKα (5831), anti-phospho-AMPKα (T172) (2535) antibodies were purchased from Cell Signaling Technology. Anti-GAPDH (G-9) (sc-365062), anti-ASC (sc-271054) and anti-ACTB/β-actin (sc-47778) were purchased from Santa Cruz Biotechnology. Anti-TFEB (A303-673A) was purchased from Bethyl Laboratories. Anti-APP (51-2700), Alexa Fluor 488 goat anti-mouse IgG (A-11001), Alexa Fluor 488 goat anti-rabbit IgG (A-11008), Alexa Fluor 594 goat anti-mouse IgG (A-11005), Alexa Fluor 594 goat anti-rabbit IgG (A-11012) were purchased from Thermo Fisher Scientific. Anti-LAMP1 (ab24170), anti-p-RELA/NF-κB-p65 (S536) (ab86299), anti-CASP1 (ab179515), anti-IL1B (ab9722) anti-SQSTM1 (ab109012) and anti-CTSD (ab75852) was purchased from Abcam. Anti-Aβ (1–16) (clone 6E10; 803017) was purchased from Biolegend. HRP-conjugated goat anti-mouse (115–035-003) and goat anti-rabbit (111-035-003) secondary antibodies were purchased from Jackson ImmunoResearch. Anti-NLRP3 (NBP2-12446) was purchased from Novus Biologicals.

### Animals

Male 5xFAD mice (Stock No: 008730, Jackson Laboratory) were obtained from Shenzhen Center for Disease Control and Prevention (Shenzhen, China) and maintained at 23 ± 2 °C and 60 ± 15% relative humidity with free access to feed and water. All mice used in the study were backcrossed to the C57BL/6 genetic background, and the hemizygous offspring at the age of 6–7 months were genotyped to select mice with gene mutations (*APP* KM670/671NL (Swedish), *APP* I716V (Florida), *APP* V717I (London), *PSEN1* M146L (A > C), *PSEN1* L286V). All animal care and experimental procedures were approved by the Animals Care and Use Committee of Guangzhou University of Chinese Medicine, in accordance with the *ARRIVE guidelines* and the *Guide for the Care and Use of Laboratory Animals* recommended by National Institutes of Health.

### Electroacupuncture treatment

Electroacupuncture (EA) was given at GV24 and bilateral GB13 acupuncture points according to the protocols described in our previous study [28, 29]. GV24 is located 1.3 mm directly above the midpoint of the mouse’s eyes, and GB13 is located 2 mm bilateral to GV24. Both of the acupoints are in the scalp of the frontal pole, which are anatomically corresponding to acupuncture points in human for treatment of cognitive disorders. For acupoints stimulation, mice were anesthetized with 2% of isoflurane (RWD Medical Co., Shenzhen, China) and positioned on a stereotaxic frame (RWD Medical Co., Shenzhen, China). Stainless-steel needles (0.16 mm in diameter and 7 mm in length, Beijing Zhongyan Taihe Medical Instrument Co. Ltd, Beijing, China) were inserted horizontally to a depth of 6 mm at the points and stimulated for 15 min at stimulus of 0.3 mA in current intensity and 2 Hz in frequency using an electrical stimulator (Hwato Medical Co., Jiangsu, China). The EA treatment was performed 5 times per week for 4 weeks.

### AAV-sh-*Tfeb* hippocampal injection

*Tfeb* shRNA sequence (CCGGCGGCAGTACTATGACTATGATCTCGAG- ATCATAGTCATAGTACTGCCGTTTTTG) is synthesized according to our previous study [22]. The non-fused viral constructs AAV-U6-shRNA (Scramble)-CMV-EGFP-SV40pA (4.16E + 12vg/ml) and AAV-U6-shRNA (*Tfeb*)-CMV-EGFP-SV40pA (2.13E + 12vg/ml) were prepared by BrainVTA (Wuhan) Co., Ltd. (Wuhan, China). 5xFAD mice (7–8 months old, male, n = 40) were injected with the viral constructs in bilateral hippocampal CA1 regions. The injection points are located in − 2.06 mm anteroposterior, ± 1.50 mm mediolateral to the bregma, and 1.4 mm below the dura [30]. The optimal viral volume of AAV-sh-*Tfeb* for each mouse was set to 2 μL, and the injection rate was set to 4 nL/sec. A detailed manipulation process was described in our previous research [28].

### Morris water maze

Morris water maze (MWM) was used to measure the hippocampus-dependent spatial memory [31]. A video analysis system (Shanghai Jiliang Software Technology Co., Ltd., Shanghai, China) was used to observe and record the swimming pattern of each mouse. The pool was filled with water (20 ± 1 ºC), with an escape platform (4.5 cm in diameter) placed 0.8–1 cm below the water surface. Mice were trained to navigate a direct path to the hidden platform when started from different, random locations around the perimeter of the tank. Any animal could not find the platform within 60 s would be placed on the platform and trained for 10–15 s. This training session was performed with 4 trials per day (the 60 s/trial, 30 min inter-trial intervals) and last for 5 days. On the sixth day, the probe test was administered. The platform was removed from the pool, and the mouse was allowed to swim freely for 1 min. The distance spent in the target quadrant compared and the platforms crossed were recorded for testing the spatial learning memory.

### Tissue extraction and Western blot analysis

Hippocampus (HI) and prefrontal cortex (PFC) were dissected and homogenized in RIPA buffer (1% Trion X-100, 1% sodium deoxycholate, 0.1% SDS) containing protease and phosphatase protease inhibitor mixture (Invitrogen, A32955 and A32957). For Aβ extraction, tissues were solubilized in RIPA buffer containing 2% SDS. Lysates were sonicated on ice and centrifuged at 150,000 rpm for 30 min. Cytosolic and nuclear proteins were extracted using the Nuclear and Cytoplasmic Protein Extraction Kit (Beyotime, P0028). After BCA assay, 10–20 μg of total protein aliquots were resolved on 8–12% Tris–acetate SDS–polyacrylamide gradient gels (Beyotime Biotechnology) and then transferred to polyvinylidene fluoride membranes. The membranes were blocked with 5% (w/v) skim milk in TBS with 1% Tween 20 for 1 h and then incubated overnight with primary antibodies in the 1% BSA blocking buffer at 4 °C. Anti-rabbit or anti-mouse secondary antibodies were applied at room temperature for 1 h. Signal intensities were detected by using ECL kits (Thermo Fisher Scientific, #34577) and quantified via ImageJ software.

### Immunohistochemistry

Anesthetized mice were perfused with 70 ml of ice-cold PBS followed by 20 ml of 4% paraformaldehyde (PFA) in PBS. After brain perfusion, tissues were fixed in 4% PFA at 4 °C for overnight. Frozen brain blocks were cut into 40-μm-thick sections on a microtome (HM 525, Leica Biosystems). After being washed with PBS for 30 min (10 min per time for 3 times), sections were permeabilized with 0.3% Triton-X 100 and 5% donkey serum in PBS at room temperature for 1.5 h. Cryosections were then incubated at 4 °C overnight with primary antibodies targeting NLRP3, ASC, SQSTM1/p62, or TFE3 at 1:300 or 1:500 solutions. After washing with PBS for 30 min (10 min per time for 3 times), Alexa Fluor-conjugated secondary antibodies (1:1500, Thermo Fisher Scientific) were applied for 1.5 h in the dark at room temperature, followed by Hoechst 33258 for nucleus staining. The slices were mounted and visualized using a Nikon A1R confocal microscope equipped with NIS-Elements Viewer 4.50 (Nikon Instruments Inc.). Fluorescence images were processed using Adobe Photoshop CS (SanJose, CA, USA). Quantification was done via ImageJ (NIH, USA).

### Statistical analysis

All statistical analysis was performed using GraphPad Prism 9.0.3. All data were presented as mean ± SEM. Statistical parameters, including the sample size (*n*) and *p* values, are reported in the figure legends. One-way ANOVA and Dunnett’s multiple comparisons test or unpaired *t*-test were performed where appropriate. Outliers were identified using the ROUT method with *Q* = 1%. A probability value of *p* < 0.05, *p* < 0.01, and *p* < 0.001 was considered to be statistically significant.

## Results

### TNEA improves spatial learning memory and reduces APP/CTFs in 5xFAD mice depending on GB13/GV24 acupoints combination

**Fig. 1:**
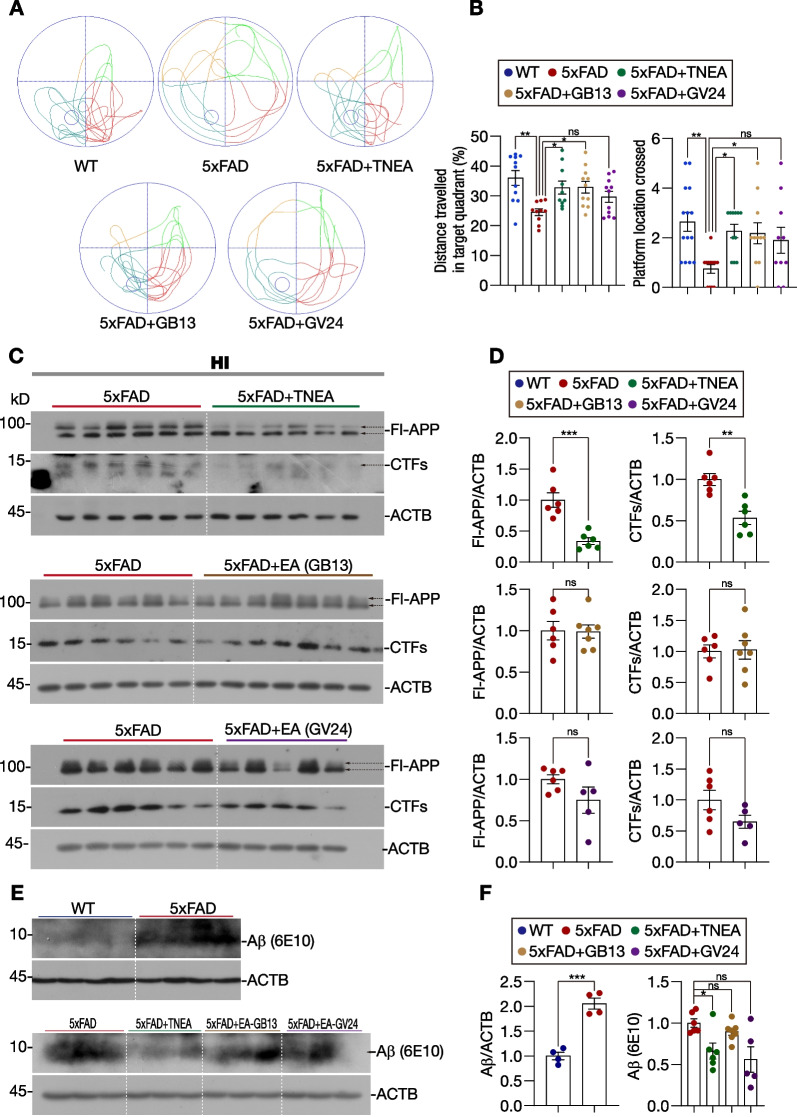
Effects of TNEA and its composing acupoints on spatial learning memory and the levels of APP/CTFs in 5xFAD mice. **A** Representative moving patterns of mice in the probe test. **B** Quantification of the proportion of the distance traveled in the target quadrant and platform location crossed (mean ± SEM, *n* = 10–14). *p < 0.05, **p < 0.01, ns (not significant, *p* > 0.05) *vs.* 5xFAD group, analyzed by one-way ANOVA. **C** Representative Western blots showed the levels of full-length APP (Fl-APP) and carboxy-terminal fragments (CTFs) in the hippocampi (HI) of mice from each group. **D** Data are quantified as mean ± SEM (male, *n* = 5–7). **p < 0.01, ***p < 0.001, ns (not significant, *p* > 0.05) *vs.* 5xFAD group, analyzed by analyzed by unpaired *t*-test. **E** Representative Western blots showed the levels of Aβ (~ 5 kDa, detected by 6E10 antibody) in the HI of mice from each group. Another batch of blots used for quantification is shown in Additional file 1: Fig. S1C. **F** Data are quantified as mean ± SEM (male, *n* = 4–7). *p < 0.05, ***p < 0.001, ns *vs.* 5xFAD group, analyzed by unpaired *t*-test and one-way ANOVA

Previously we had demonstrated the cognitive-enhancing effects of TNEA therapy, which electrically stimulates the GV24 and bilateral GB13 acupoints located on the forebrain, in 5xFAD mice [28]. The emerging question is whether the individual acupoints need to be combined to exert the observed effect. Therefore, we compared the effects of EA on the single and combined acupoints on the spatial memory in 5xFAD mice using the Morris water maze (MWM). Consistent with our previous finding, the mice treated with TNEA showed improved preference to the target quadrant in the probe test, as quantified by the percentage of distance traveled in the target quadrant and platform location crossed (Fig. 1A and B). Interestingly, EA on the single acupoint GB13, but not GV24, showed comparable improvement on the spatial memory (Fig. 1A and B). Since the memory impairment in 5xFAD mice is mainly associated with amyloid precursor protein (APP) overexpression, we then compared the effects of TNEA and its composing acupoints on the reduction of APP and its C-terminal fragments (CTFs). Consistent with the results from our previous study [28], TNEA treatment reduced the levels of full-length (Fl)-APP and/or CTFs in the hippocampus (HI) (Fig. 1C and D) and prefrontal cortex (PFC) (Additional file 1: Fig. S1A and B). By contrast, neither GB13-EA nor GV24-EA had effects on the levels of Fl-APP and CTFs in the HI (Fig. 1C and D). Furthermore, the levels of Aβ (detected by 6E10 antibody) in the HI of 5xFAD mice were significantly reduced by TNEA, but not GB13-EA or GV24-EA (Fig. 1E and F; Additional file 1: Fig. S1C). In the PFC, GB13-EA (but not GV24-EA) also reduced CTFs (*p* = *0.0017*), although the reduction was not less dramatic as that of TNEA (*p* = *0.0004*) (Additional file 1: Fig. S1A and B). Together, these results suggest that the combination of GB13 and GV24 points in the TNEA therapy may synergistically reduce the APP/CTFs levels via autophagic degradation [28] and other unknown mechanisms, thus leading to the attenuation of memory impairment in 5xFAD mice.

### Acupoints combination in TNEA synergistically promotes the degradation of NLRP3 inflammasome components via TFEB-mediated autophagy-lysosomal pathway

**Fig. 2:**
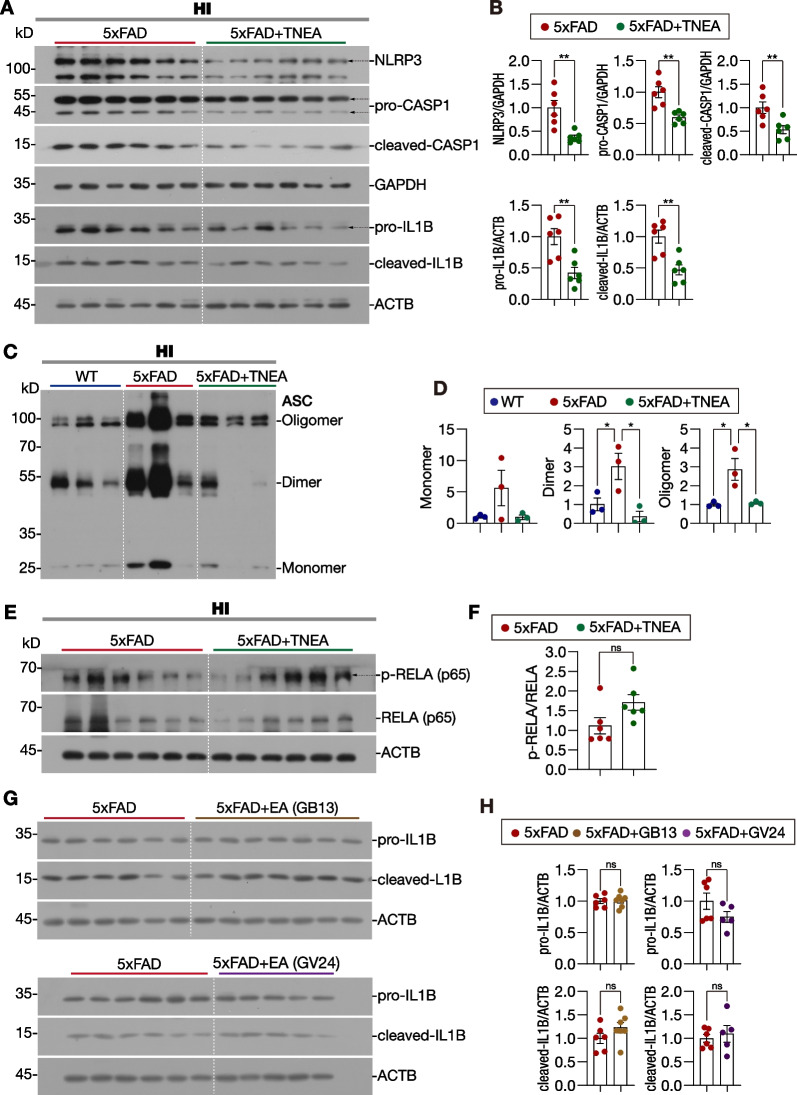
TNEA promotes the degradation of NLRP3 inflammasome components in the hippocampi of 5xFAD mice. **A** Representative Western blots showed the levels of NLRP3, CASP1, and IL1B in the HI of mice from each group. **B** Data are quantified as mean ± SEM (male, *n* = 6). **p < 0.01 *vs.* 5xFAD group analyzed by unpaired *t*-test or unpaired *t* test with Welch's correction. **C** Representative Western blots showed the levels of ASC in the HI of mice from each group. **D** Data are quantified as mean ± SEM (male, *n* = 3). *p < 0.05 *vs.* 5xFAD group analyzed by one-way ANOVA. **E** Representative Western blots showed the levels of p-RELA (p65) and RELA (p65) in the HI of mice from each group. **F** Data are quantified as mean ± SEM (male, *n* = 6). ns *vs.* 5xFAD group analyzed by unpaired *t*-test. **G** Representative Western blots showed the levels of pro-IL1B and cleaved IL1B expression in the HI of mice from each group. **H** Data are quantified as mean ± SEM (male, *n* = 5–7). *p < 0.05, ns *vs.* 5xFAD group analyzed by unpaired *t*-test

**Fig. 3:**
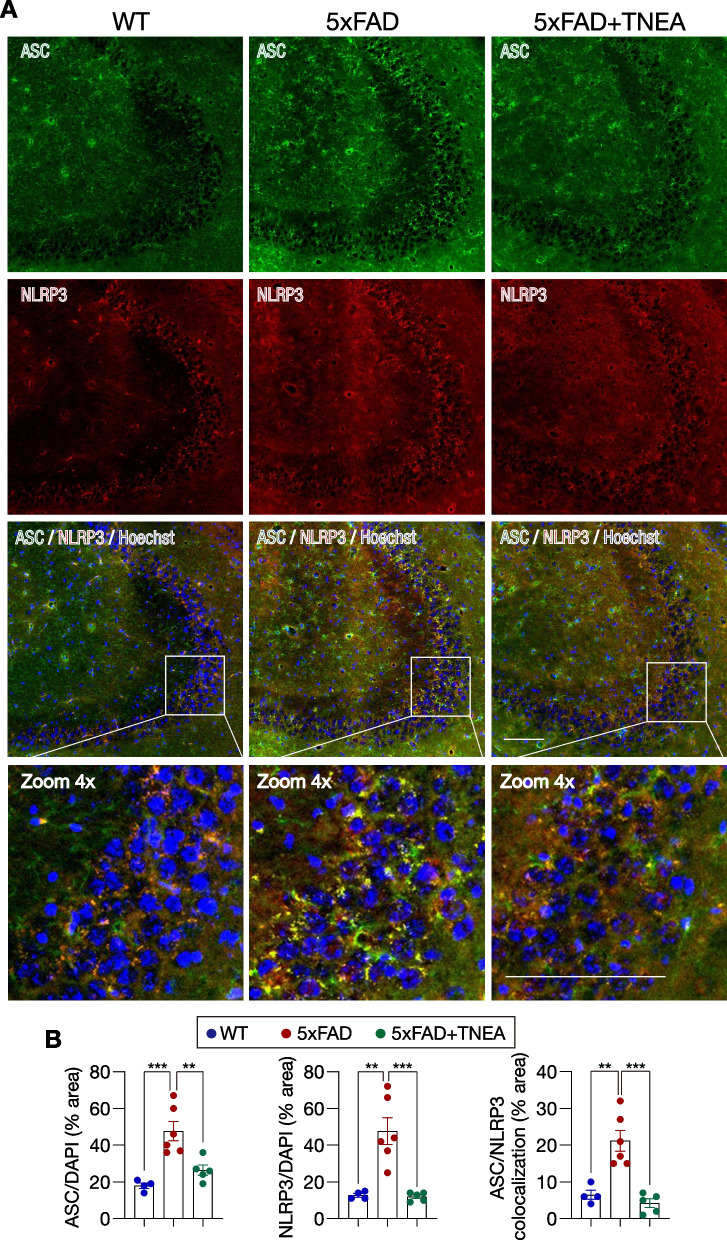
Effects of TNEA on NLRP3–ASC complex in the hippocampi of 5xFAD mice. **A** Representative immunohistochemical (IHC) staining images of NLRP3 (red), ASC (green), nuclei (Hoechst, blue) and their colocalization in the hippocampal CA3 of mice from each group. Scale bar: 100 μm. 4× magnified images were shown for the selected area. Negative controls are shown in Additional file 1: Fig. S3. **B** Images were processed using ImageJ to quantify the average area of NLRP3, ASC and their colocalization (divided by the area of Hoechst). Data are quantified as mean ± SEM (male, *n* = 4–6) and analyzed by one-way ANOVA. **p < 0.01, ***p < 0.001 *vs.* 5xFAD group

Previous studies have demonstrated the activation of NLRP3 inflammasome and elevation of IL1B in the brains of AD patients and AD transgenic mice, such as the APP/PS1 and 5xFAD mice [3, 4, 32, 33]. Based on our previous findings that TNEA reduced Aβ load and inhibited microglia activation in the brains of 5xFAD mice [28], herein we determined whether and how TNEA inhibits Aβ-induced NLRP3 inflammasome activation. Firstly, we confirmed the activation of NLRP3 inflammasome accompanied with overexpression of APP in 5xFAD mouse brain, as evidenced by the increased levels of phosphorylated (p-)NF-κB-p65 (officially termed as RELA), NLRP3, pro-/cleaved-CASP1, and pro-/cleaved IL1B, and overexpressed Fl-APP/CTFs in the HI from mice aged 8 months (Additional file 1: Fig. S2A and B) and 13 months (Additional file 1: Fig. S2C and D). TNEA treatment significantly reduced the levels of NLRP3, pro-/cleaved-CASP1, and pro-/cleaved IL1B in the HI of 5xFAD mice (Fig. 2A and B). Meanwhile, the dimers and oligomers of ASC, which indicate the formation of NLRP3–ASC complex, were reduced in the hippocampi of 5xFAD mice treated with TNEA (Fig. 2C and D). The immunohistochemistry (IHC) results further confirmed that TNEA reduced NLRP3–ASC oligomers, evidenced by the decrease in the area of NLRP3, ASC and NLRP3/ASC colocalization in the CA3 region of HI of 5xFAD mice treated with TNEA (Fig. 3, negative controls are shown in Additional file 1: Fig. S3). Notably, TENA did not affect the levels of p-RELA (Fig. 2E and F), indicating that TNEA may not inhibit the production of NLRP3 and pro-IL1B mediated by RELA activation [34]. Then we wondered whether the acupoints combination is necessary for TNEA to reduce NLRP3 inflammasome. The results showed that neither GB13-EA nor GV24-EA reduced the level of pro-/cleaved IL1B (Fig. 2G and H), indicating the synergetic effect of GB13 and GV24 combination in inhibiting NLRP3 inflammasome.

**Fig. 4:**
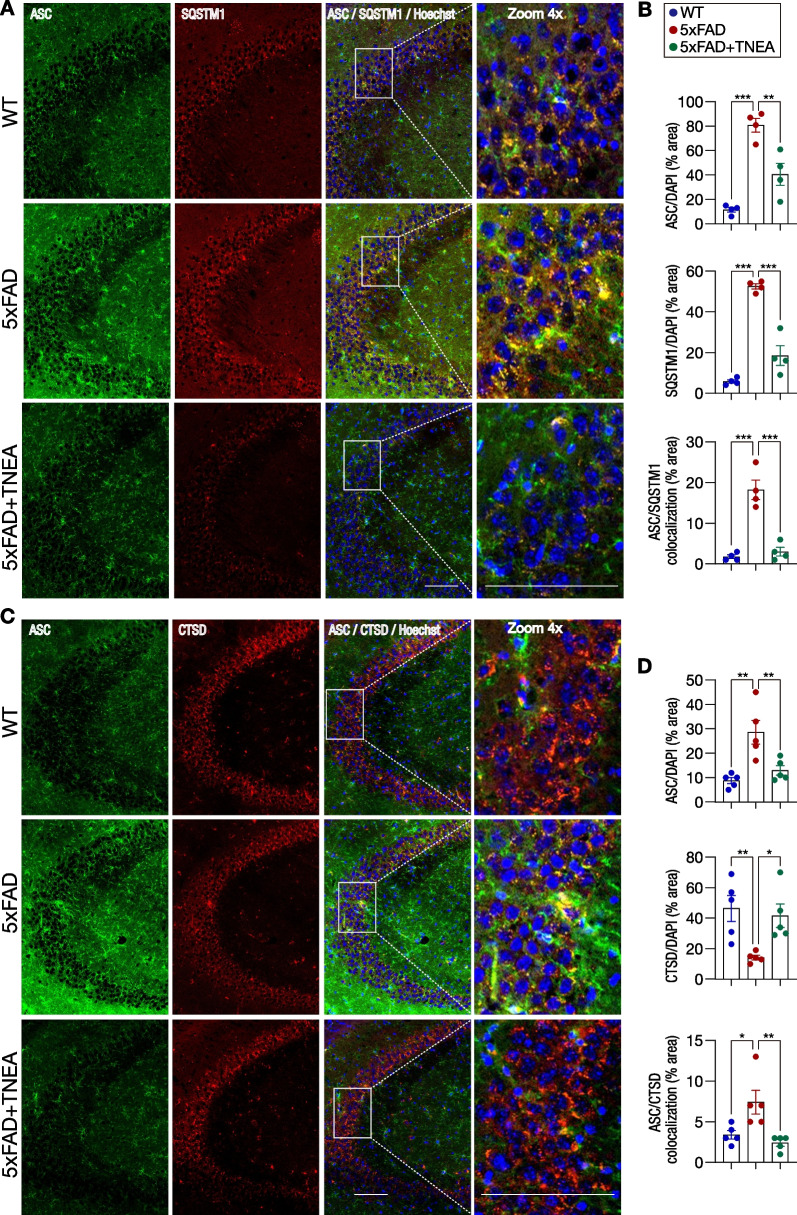
Effects of TNEA on the autophagic recognition/degradation of ASC specks in the hippocampi of 5xFAD mice. **A** Representative IHC staining images of ASC (green), SQSTM1/p62 (red), nuclei (Hoechst, blue) and their colocalization in the hippocampal CA3 of mice from each group. Scale bar: 100 μm. 4× magnified images were shown for the selected area. Negative controls are shown in Additional file 1: Fig. S3. **B** Images were processed using ImageJ to quantify the average area of ASC, SQSTM1 and their colocalization (divided by the area of Hoechst). Data are quantified as mean ± SEM (male, *n* = 4) and analyzed by one-way ANOVA. **p < 0.01, ***p < 0.001 *vs.* 5xFAD group. **C** Representative IHC staining images of ASC (green), cathepsin D (CTSD, red), nuclei (Hoechst, blue) and their colocalization in the hippocampal CA3 of mice from each group. Scale bar: 100 μm. 4× magnified images were shown for the selected area. Negative controls are shown in Additional file 1: Fig. S3. **D** Images were processed using ImageJ to quantify the average area of ASC, CTSD and their colocalization (divided by the area of Hoechst). Data are quantified as mean ± SEM (male, *n* = 5) and analyzed by one-way ANOVA. *p < 0.05, **p < 0.01 *vs.* 5xFAD group

Since the inflammasome components NLRP3 and ASC, and pro-IL1B can be degraded by ALP [35–37], we next addressed whether TNEA reduces NLRP3 inflammasome components via autophagic degradation, by double staining of ASC with either sequestosome 1 (SQSTM1/p62, an autophagy substrate) or cathepsin D (CTSD, a lysosomal protease) in the HI of 5xFAD mice. For autophagic degradation, the polyubiquitinated ASC aggregates need to be recruited by SQSTM1 and delivered to autophagosomes [36]. The results showed that large ASC specks were colocalized with SQSTM1 punctae in the CA3 region of HI from 5xFAD mice (Fig. 4A and B, negative controls are shown in Additional file 1: Fig. S3), indicating the accumulation of undigested ASC–SQSTM1 aggregates due to deficient autophagic degradation. TNEA treatment significantly reduced the positive signals of ASC, SQSTM1, and their colocalized complex, which suggests that TNEA promotes the autophagic recognition and degradation of ASC oligomers via SQSTM1 (Fig. 4A and B). Furthermore, the results from ASC/CTSD co-staining demonstrated that the average area of CTSD was decreased in the CA3 region of 5xFAD mice, indicating deficient lysosomal activity (Fig. 4C and D, negative controls are shown in Additional file 1: Fig. S3). Notably, large punctae of CTSD were colocalized with ASC specks in the CA3 region of 5xFAD mice (Fig. 4C and D), which suggests that the undegraded ASC specks positive for inactive CTSD in the lysosomes. TNEA treatment significantly reduced the area of ASC specks and ASC/CTSD colocalization and increased the level of CTSD (Fig. 4C and D). Together, these results suggest that TNEA rescues the deficient autophagy flux, thus promoting the autophagic recognition and lysosomal degradation of NLRP3 inflammasome components in the HI of 5xFAD mice.

**Fig. 5:**
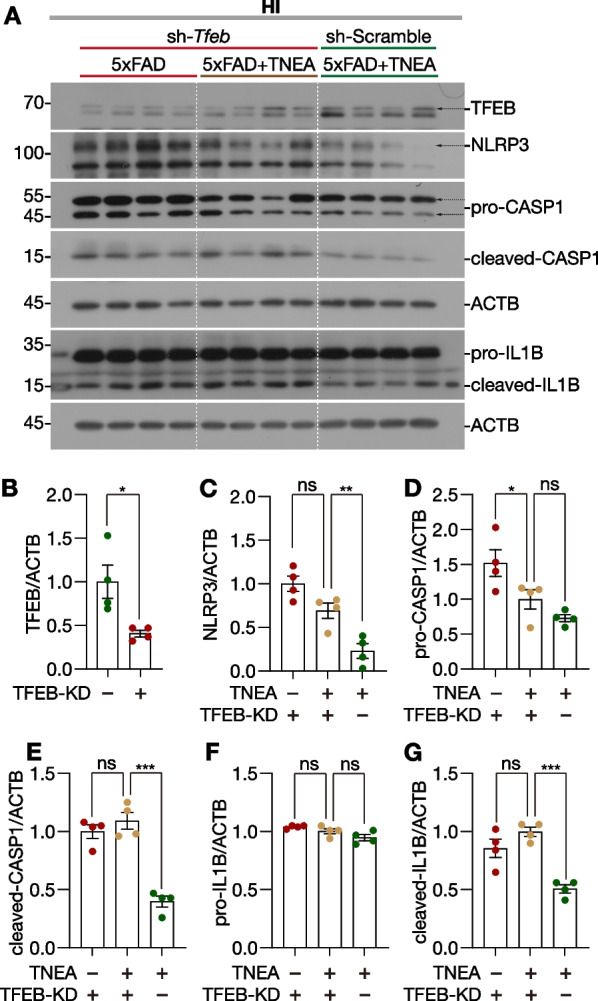
TNEA promotes the autophagic degradation of NLRP3 inflammasome components depending on TFEB. **A** Representative Western blots showed the levels of TFEB, NLRP3, CASP1 and IL1B in the hippocampus of mice from each group with/without *Tfeb* knockdown (KD). **B**–**G** Data are quantified as mean ± SEM (male, *n* = 4). *p < 0.05, **p < 0.01, ***p < 0.001, ns (*p* > 0.05) *vs.* TNEA^+^-TFEB-KD^+^ group, analyzed by unpaired t test (TFEB-KD^+^ *vs.* TFEB-KD^−^) and one-way ANOVA

Previously we had proved that TNEA promoted autophagy flux via activating TFEB in 5xFAD mice [28]. Therefore, we wondered whether TFEB is necessary for the degradation of NLRP3 inflammasome by TNEA treatment. For this, adeno-associated virus (AAV) vectors carrying small hairpin RNA (shRNA) targeting *Tfeb* or Scramble were injected into the bilateral hippocampal CA1 regions of 5xFAD mice 2 weeks before TNEA treatment. The knockdown (KD) efficiency was ~ 50% as quantified by the protein level of TFEB determined by Western blot (Fig. 5A and B). In mice with TFEB-KD, TNEA treatment failed to reduce the levels of NLRP3 (Fig. 5A and C), pro-/cleaved-IL1B (Fig. 5A, F, G) and cleaved-CASP1 (Fig. 5A and E). Notably, the reduction of pro-CASP1 by TNEA was not blocked by TFEB-KD (Fig. 5A and D), indicating that TFEB-mediated ALP is not responsible for pro-CASP1 turnover. When comparing the TNEA groups with or without TFEB-KD, the results showed that TFEB-KD blocked the effects of TNEA on reducing NLRP3 (Fig. 5A and C), cleaved-CASP1 (Fig. 5A and E) and cleaved-IL1B (Fig. 5A and G), but not pro-CASP1 (Fig. 5A and D) and pro-IL1B (Fig. 5A and F). These results indicate that TFEB-KD partially blocked the degradation of NLRP3 inflammasome components by TNEA treatment.

**Fig. 6:**
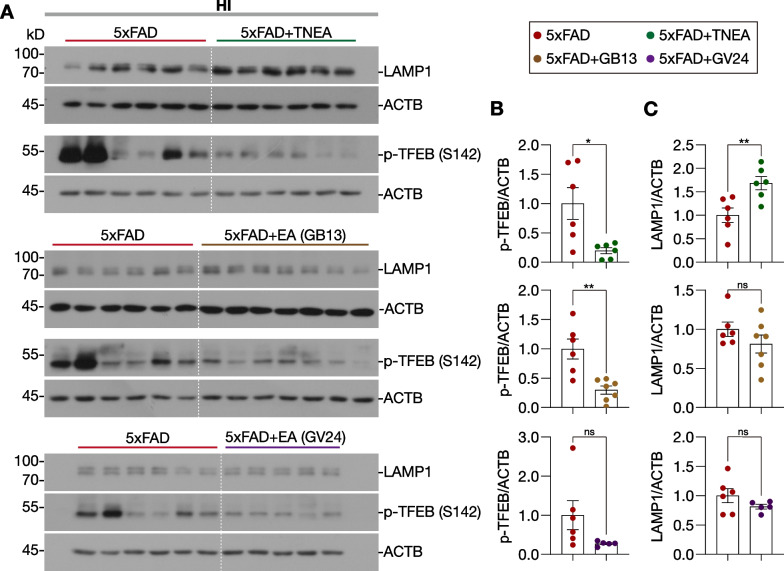
Effects of TNEA and its composing acupoints on TFEB activation and lysosomal biogenesis in the hippocampi of 5xFAD mice. **A** Representative Western blots showed the levels of phosphorylated (p-) TFEB (S142) and the lysosome marker LAMP1 in the hippocampi of mice from each group. **B**, **C** Data are quantified as mean ± SEM (male, *n* = 5–7). *p < 0.05, **p < 0.01, ns (*p* > 0.05) *vs.* 5xFAD group analyzed by unpaired *t*-test or unpaired *t*-test with Welch's correction

Then we determined whether the acupoints combination is necessary for TNEA to activate TFEB and lysosomal biogenesis. In the HI, GB13-EA (but not GV24-EA) activated TFEB as TNEA did, indicated by the significant decrease in p-TFEB (S142) (Fig. 6A and B). However, EA on individual GB13 or GV24 cannot increase the level of LAMP1 (Fig. 6A and C). In addition, EA on individual GB13 or GV24 was not sufficient to activate TFEB in the PFC (Additional file 1: Fig. S4). Together, these data indicate that EA on GB13 or GV24 alone may be not sufficient to activate TFEB in both HI and PFC to promote lysosomal biogenesis responsible for the efficient degradation of NLRP3 inflammasome components in the HI of 5xFAD mice.

### TNEA and its composing acupoints activate TFE3 in the hippocampus of 5xFAD mice

**Fig. 7:**
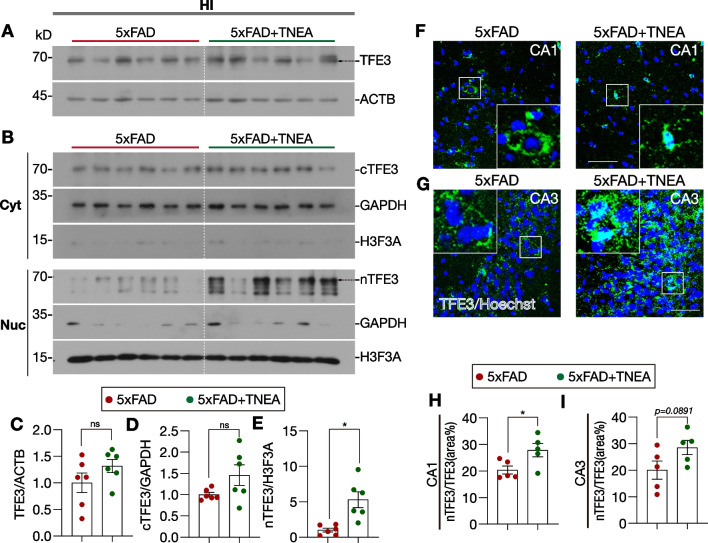
TNEA activates TFE3 in the hippocampi of 5xFAD mice. **A**, **B** Representative Western blots showed the levels of total TFE3 (**A**) and cytosolic (Cyt) /nuclear (Nuc) levels of TFE3 (**B**) in the hippocampi (HI) of mice from each group. GAPDH and H3F3A (H3 histone) were used as cytosolic and nuclear loading controls, respectively. **C**–**E** Quantitation results of **A** and **B**. Data are quantified as mean ± SEM (male, *n* = 6). *p < 0.05, ns (*p* > 0.05) *vs.* 5xFAD group analyzed by unpaired *t*-test or unpaired *t*-test with Welch's correction. **F**–**G** Representative IHC staining images of TFE3 (green) and nuclei (Hoechst, blue) in the hippocampal CA1 (**F**) and CA3 (**G**) of mice from each group. Scale bar: 100 μm. 4× magnified images were shown for the selected area. **H**, **I** Images were processed using ImageJ to quantify the average area of TFE3, (divided by the area of Hoechst). Data are quantified as mean ± SEM (male, *n* = 5) and analyzed by unpaired *t*-test. *p < 0.05 *vs.* 5xFAD group

Since TFE3, another MiTF/TFE transcription factor, shares similar function as TFEB in regulating ALP [17, 18], we further determined whether TNEA and its composing acupoints also activate TFE3 in the HI of 5xFAD mice. In the hippocampal lysates, TNEA treatment did not affect the total level of TFE3 (Fig. 7A and C). However, the level of TFE3 in the nuclear fraction (nTFE3) significantly increased by TNEA treatment (Fig. 7B and E). The IHC of TFE3 in the HI further demonstrated the significant increase in the percentage of nTFE3 in CA1 region, and mild (but not significant) increase (*p* = *0.0891*) of nTFE3 in CA3 region from mice treated with TNEA (Fig. 7F–I). Notably, EA on the individual acupoint GB13, but not GV24, also showed the trend of increase in nTFE3 (*p* = *0.0542*) in the HI of 5xFAD mice as determined by Western blots (Additional file 1: Fig. S5). These results indicate that activation of both TFEB and TFE3 may enhance the autophagic degradation of NLRP3 inflammasome by TNEA treatment in 5xFAD mice.

### TNEA and its composing acupoints activate AMPK and inhibit AKT in the hippocampus of 5xFAD mice

**Fig. 8:**
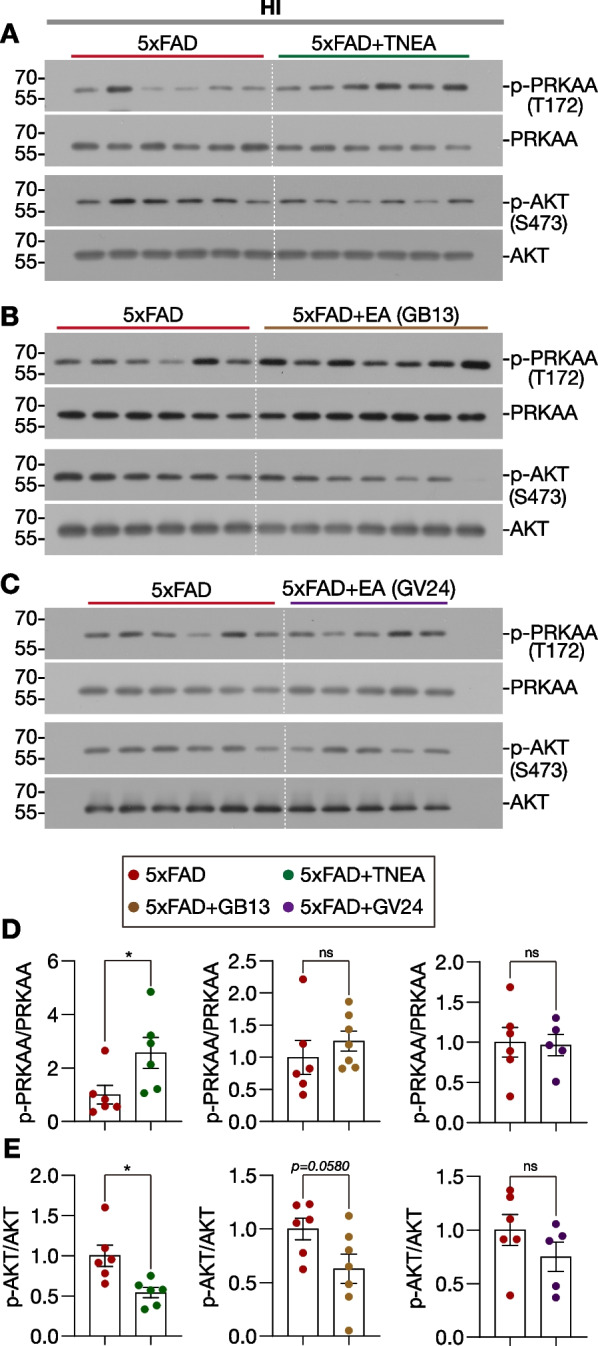
Effects of TNEA and its composing acupoints on the phosphorylation of AMPK and AKT in the hippocampi of 5xFAD mice. **A**–**C** Representative Western blots showed the levels of phosphorylated (p-)/total AMPKα (PRKAA) and AKT in the HI of mice from each group. **D**, **E** Data are quantified as mean ± SEM (male, *n* = 5–7). *p < 0.05, ns (*p* > 0.05) *vs.* 5xFAD group analyzed by unpaired *t*-test or unpaired *t*-test with Welch's correction

Previously we found that TNEA activates TFEB via inhibiting several kinases such as MTOR, MAPK1, and AKT in the HI and PFC of 5xFAD mice [28]. Since AMPK is positive regulator of ALP, acting through multiple signaling pathways including MTORC1 inhibition and TFEB/TFE3 activation [38, 39], we determined whether TNEA and its composing acupoints activate AMPK in the HI of 5xFAD mice. The results demonstrated that TNEA (Fig. 8A and D), but not the individual acupoint GB13 (Fig. 8B and D) and GV24 (Fig. 8C and D), significantly increased the level of p-AMPKα (T172) (officially termed as PRKAA), indicating that the acupoints combination are necessary for TNEA to activate AMPK. Similarly, we found that GB13 and GV24 combination synergistically inhibited p-AKT, although GB13 alone showed the trend (*p* = *0.0580*) to inhibit p-AKT (Fig. 8E).

## Discussion

**Fig. 9:**
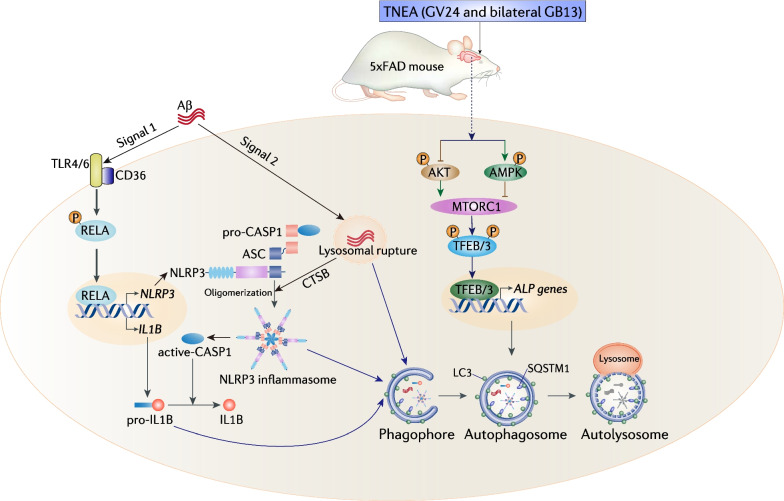
A mechanistic model illustrating that TNEA promotes TFEB/TFE3-mediated autophagic degradation of NLRP3 inflammasome components. Simultaneous EA on the combined acupoints (GV24 and bilateral GB13) synergistically modulates multiple kinases including AMPK, AKT and MTOR which act upstream of TFEB/3 to promote ALP, thus leading to efficient degradation of Aβ, NLRP3 inflammasome components and IL1B in the hippocampi of 5xFAD mice

The crosstalk between NLRP3 inflammasome activation and ALP impairment plays key roles in the Aβ pathology of AD. Aβ activates NLRP3 inflammasome via two signals: soluble Aβ is recognized by the pattern-recognition receptor CD36 and TLR4/TLR6 complex, which promotes the nuclear translocation of RELA/NF-κB responsible for the transcription of *NLRP3* and pro-*IL1B* [40] (Signal 1); phagocytosis of Aβ in the lysosomes leads to lysosome rupture and the leakage of cathepsin B (CTSD), which promotes the oligomerization of NLRP3 inflammasome complex [40, 41] (Signal 2) (Fig. 9). New evidence suggests that ALP deficiency, manifested by faulty autolysosome acidification in neurons, induces build-up of Aβ plaques in AD [12]. Therefore, promoting ALP, especially by targeting its master regulators TFEB/TFE3, may simultaneously rescue the ALP impairment, inhibit neuroinflammation and Aβ pathology, thus representing a fascinating strategy for the early prevention and treatment of AD [2, 7, 14]. Advancing our previous finding that a novel EA therapy named TNEA attenuates Aβ pathology and improves cognition in 5xFAD mice [28], here we further demonstrated that: (1) TNEA inhibits Aβ-induced activation of NLRP3 inflammasome in the hippocampus of 5xFAD mice via promoting the autophagic degradation of inflammasome components (NLRP3 and ASC) and pro-IL1B mediated by TFEB (Fig. 9); (2) TNEA activated both TFEB and TFE3 to promote ALP via regulating multiple upstreaming kinases including AMPK, AKT (Fig. 9); and (3) The acupoints GB13 and GV24 in TNEA need to be combined to efficiently modulate the molecular events leading to inhibition of NLRP3 inflammasome.

In our study, we found that TNEA activated both TFEB [28] (Fig. 6) and TFE3 (Fig. 7) in the HI of 5xFAD mice, and TFEB-KD (~ 50%) significantly blocked the autophagic degradation of NLRP3 and pro-IL1B (Fig. 5). However, whether TFEB and TFE3 plays equal or different roles in the autophagic degradation of NLRP3 inflammasome remains be to addressed. Meanwhile, a previous study reported the differential expression of TFEB and TFE3 in the nuclei of neurons and glia cells in the hippocampus, and suggested more important roles of TFEB in glia and TFE3 in neurons, respectively [20]. Therefore, determining the nuclear localization of both TFEB and TFE3 in neurons and glia cells in the HI of 5xFAD mice treated with/without TNEA will help to determine the differential roles of TFEB/TFE3 in regulating neuronal/glia ALP and NLRP3 inflammasome in our further studies. Specific knockout (KO) of TFEB/TFE3 in neurons/glia cells of HI will also be necessary for validating our findings. For the composing acupoints in TNEA, why GB13 has a more prominent effect on the activation of both TFEB and TFE3 (Fig. 6, Additional file 1: Fig. S5) compared to GV24 needs to be further investigated.

The activation of TFEB/TFE3 by TNEA is mediated by regulation of multiple upstreaming kinases (Fig. 9). Notably, the activation of AMPK may be an important event mediating the effects of TNEA, since AMPK participates in long-term modulation of autophagy and lysosomal function through multiple direct and indirect mechanisms [38]. However, how TNEA activates AMPK remains to be addressed. One possible mechanism is via activation of cannabinoid (CB) receptors, which inhibits AKT–MTORC1 pathway and activates AMPK via calcium/calmodulin-dependent protein kinase kinase 2 beta (CaMKKβ) [42, 43]. For example, EA reportedly reduced the inflammatory pain by inhibiting the activation of NLRP3 inflammasome through CB2 receptors [44]. Since CB receptors play important roles in neurological disorders including AD [45], it is worthwhile for us to determine whether and how TNEA activates brain CB receptors to regulate ALP and inhibit neuroinflammation.

In summary, our findings reveal for the first time that a combined EA therapy synergistically promote the degradation of NLRP3 inflammasome components via TFEB/3-mediated ALP, thus inhibiting Aβ pathology, neuroinflammation and improving cognition in AD mice. Further investigation on the molecular/anatomical basis of this combined acupoints therapy may lead to an efficient non-pharmacological treatment for AD.

## Supplementary Information

**Additional file 1: Figure S1.** Effects of TNEA and its composing acupoints on the levels of APP/CTFs/Aβ in 5XFAD mice. (**A**) Representative Western blots showed the levels of full-length APP (FlAPP) and carboxy-terminal fragments (CTFs) in the prefrontal cortex (PFC) of mice from each group. (**B**) Data are quantified as mean ± SEM (male, *n *= 5-7). **p<0.01, ***p<0.001, ns (not significant, *p*>0.05) vs. 5xFAD group, analyzed by unpaired *t*-test or Mann–Whitney *U* test. (**C**) Representative Western blots showed the levels of Aβ in the hippocampus (HI) of mice from each group (*n *=3). The combined quantification data are shown in Fig. 1F. **Figure S2.** Activation of NLRP3 inflammasome in the hippocampus of 5xFAD mice. (**A**, **C**) Representative Western blots showed the levels of phosphorylated (*p*-)RELA (p65), NLRP3, CASP1 and IL1B in the hippocampi (HI) of 5xFAD mice at the age of 8 months (**A**) and 13 months (**C**). (**B**, **D**) Data are quantified as mean ± SEM (male, *n* = 6). *p<0.05, **p<0.01, ***p<0.001, ns (not significant, *p*>0.05) vs. WT group, analyzed by unpaired *t*-test, unpaired *t*-test with Welch's correction, or Mann–Whitney *U* test. **Figure S3.** Negative controls for the IHC of ASC, NLRP3, SQSTM1 and CTSD. The slides from each group were stained with each 1st antibody at the same dilution as performed in Fig. 3 and 4, but without 2nd antibodies; or stained with 2nd antibodies at the same dilution as performed in Fig. 3 and 4, but without 1st antibodies. Images were visualized at the same settings as performed in Fig. 3 and 4. Scale bar: 100 μm. **Figure S4.** Effects of TNEA and its composing acupoints on TFEB activation in the prefrontal cortex of 5XFAD mice. (**A**) Representative Western blots showed the levels of phosphorylated (*p*-) TFEB (S142) in the prefrontal cortex (PFC) of mice from each group. (**B**) Data are quantified as mean ± SEM (male, *n* = 5-7). **p<0.01, ns (*p*>0.05) vs. 5xFAD group analyzed by unpaired *t*-test, unpaired *t*-test with Welch's correction, or Mann–Whitney U test. **Figure S5.** Effects of the composing acupoints of TNEA on TFE3 activation in the hippocampi of 5xFAD mice. (**A**) Representative Western blots showed the levels of cytosolic (Cyt) /nuclear (Nuc) levels of TFE3 in the hippocampi (HI) of mice from each group. GAPDH and H3F3A (H3 histone) were used as cytosolic and nuclear loading controls, respectively. (**B**) Data were quantified as mean ± SEM (male, *n*=5-7). *p<0.05, **p<0.01, ns (*p*>0.05) vs. 5xFAD group analyzed by unpaired *t*-test. **Figure S6.** Unprocessed scans of all immunoblots.

**Additional file 2. Table S1.** Source data and statistical analysis.

## Data Availability

All data are available in the main text or the supplementary materials. Uncropped/unprocessed scans of all immunoblots and statistical source data in the paper are included as Additional file 1: Fig. S6 and Additional file 2: Table S1, respectively. Other information that supports the findings of this study is available from the corresponding author upon reasonable request.

## Abbreviations

AD: Alzheimer’s disease
Aβ: β-Amyloid
APP: β-Amyloid precursor protein
ALP: Autophagy-lysosomal pathway
AMPK: AMP-activated protein kinase
AKT: AKT serine/threonine kinase 1
CTFs: C-terminal fragments
CTSD: Cathepsin D
Cyt: Cytosolic
EA: Electroacupuncture
Nuc: Nuclear
TFEB: Transcription factor EB
TFE3: Transcription factor binding to IGHM enhancer 3
PFC: Prefrontal cortex
HI: Hippocampus
LAMP1: Lysosomal-associated membrane protein 1
MWM: Morris water maze
SQSTM1/p62: Sequestosome 1
