# Supplementary material for: TNEA therapy promotes the autophagic degradation of NLRP3 inflammasome in a transgenic mouse model of Alzheimer’s disease via TFEB/TFE3 activation
Source: J Neuroinflammation. 2023 Feb 2;20:21. doi: 10.1186/s12974-023-02698-w (PMC9896717; doi:10.1186/s12974-023-02698-w)
Supplement: Supplementary file 1 — Additional file 1: Figure S1. Effects of TNEA and its composing acupoints on the levels of APP/CTFs/Aβ in 5XFAD mice. (A) Representative Western blots showed the levels of full-length APP (FlAPP) and carboxy-terminal fragments (CTFs) in the prefrontal cortex (PFC) of mice from each group. (B) Data are quantified as mean ± SEM (male, n = 5-7). **p<0.01, ***p<0.001, ns (not significant, p>0.05) vs. 5xFAD group, analyzed by unpaired t-test or Mann–Whitney U test. (C) Representative Western blots showed the levels of Aβ in the hippocampus (HI) of mice from each group (n =3). The combined quantification data are shown in Fig. 1F. Figure S2. Activation of NLRP3 inflammasome in the hippocampus of 5xFAD mice. (A, C) Representative Western blots showed the levels of phosphorylated (p-)RELA (p65), NLRP3, CASP1 and IL1B in the hippocampi (HI) of 5xFAD mice at the age of 8 months (A) and 13 months (C). (B, D) Data are quantified as mean ± SEM (male, n = 6). *p<0.05, **p<0.01, ***p<0.001, ns (not significant, p>0.05) vs. WT group, analyzed by unpaired t-test, unpaired t-test with Welch's correction, or Mann–Whitney U test. Figure S3. Negative controls for the IHC of ASC, NLRP3, SQSTM1 and CTSD. The slides from each group were stained with each 1st antibody at the same dilution as performed in Fig. 3 and 4, but without 2nd antibodies; or stained with 2nd antibodies at the same dilution as performed in Fig. 3 and 4, but without 1st antibodies. Images were visualized at the same settings as performed in Fig. 3 and 4. Scale bar: 100 μm. Figure S4. Effects of TNEA and its composing acupoints on TFEB activation in the prefrontal cortex of 5XFAD mice. (A) Representative Western blots showed the levels of phosphorylated (p-) TFEB (S142) in the prefrontal cortex (PFC) of mice from each group. (B) Data are quantified as mean ± SEM (male, n = 5-7). **p<0.01, ns (p>0.05) vs. 5xFAD group analyzed by unpaired t-test, unpaired t-test with Welch's correction, or Mann–Whitney U test. Figure S5. [file 12974_2023_2698_MOESM1_ESM.pdf]

Additional file for

TNEA therapy promotes the autophagic degradation of NLRP3 inflammasome in a transgenic mouse model of Alzheimer's disease via TFEB/TFE3 activation

Wenjia Lin[1,2], Zhao Li[1], Guangfeng Liang[1], Runjin Zhou[1], Xiaoyan Zheng[1,3], Rongrong Tao[1], Qingwei Huo[1], Chengfu Su[4], Min Li[4], Nenggui Xu[1]*, Chunzhi Tang[1]*, Ju-Xian Song[1,4]*

*Corresponding author. Email: juxian.song@gmail.com

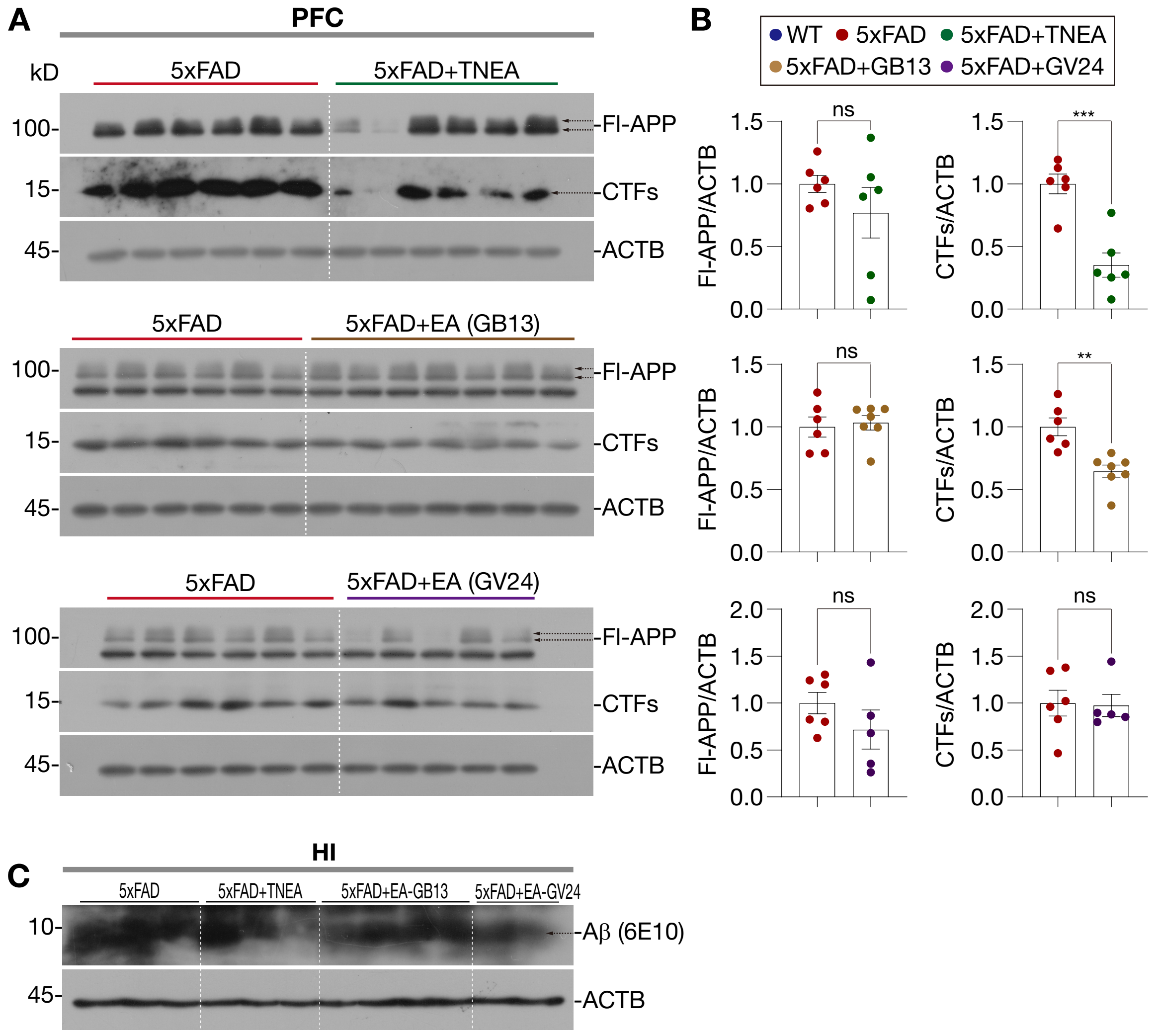

Fig. S1. Effects of TNEA and its composing acupoints on the levels of APP/CTFs/Aβ in 5XFAD mice. (**A**) Representative Western blots showed the levels of full-length APP (Fl-APP) and carboxy-terminal fragments (CTFs) in the prefrontal cortex (PFC) of mice from each group. (**B**) Data are quantified as mean ± SEM (male, n = 5-7). **p<0.01, ***p<0.001, ns (not significant, p>0.05) vs. 5xFAD group, analyzed by unpaired t-test or Mann-Whitney U test. (**C**) Representative Western blots showed the levels of Aβ in the hippocampus (HI) of mice from each group (n =3). The combined quantification data were shown in Fig. 1F.

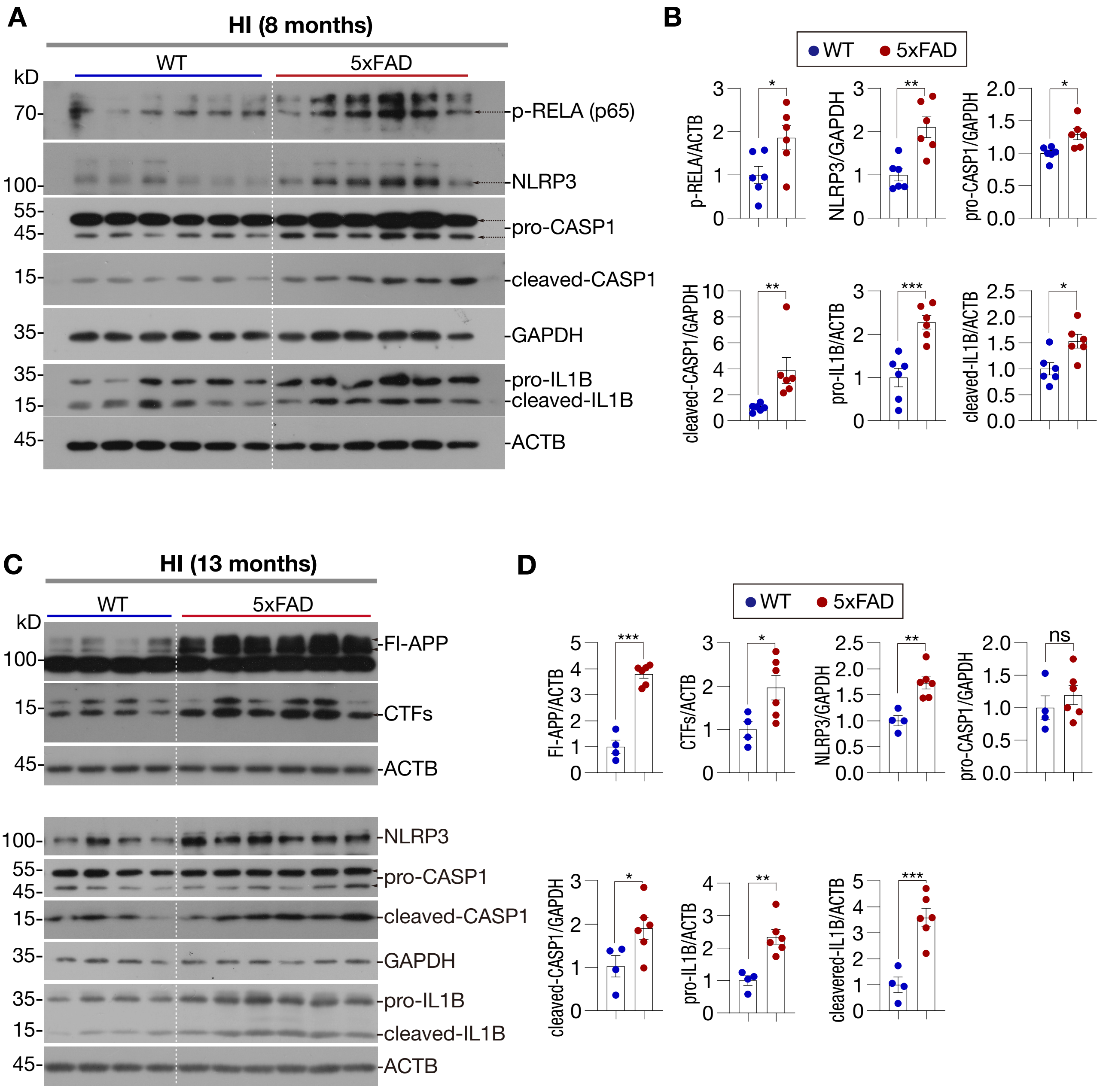

Fig. S2. Activation of NLRP3 inflammasome in the hippocampus of 5xFAD mice. (**A**, **C**) Representative Western blots showed the levels of phosphorylated (*p*-)RELA (p65), NLRP3, CASP1 and IL1B in the hippocampi (HI) of 5xFAD mice at the age of 8 months (**A**) and 13 months (**C**). (**B**, **D**) Data are quantified as mean ± SEM (male, *n* = 6). **p*<0.05, ***p*<0.01, ****p*<0.001, ns (not significant, *p*>0.05) vs. WT group, analyzed by unpaired t-test, unpaired t-test with Welch's correction, or Mann Whitney U test

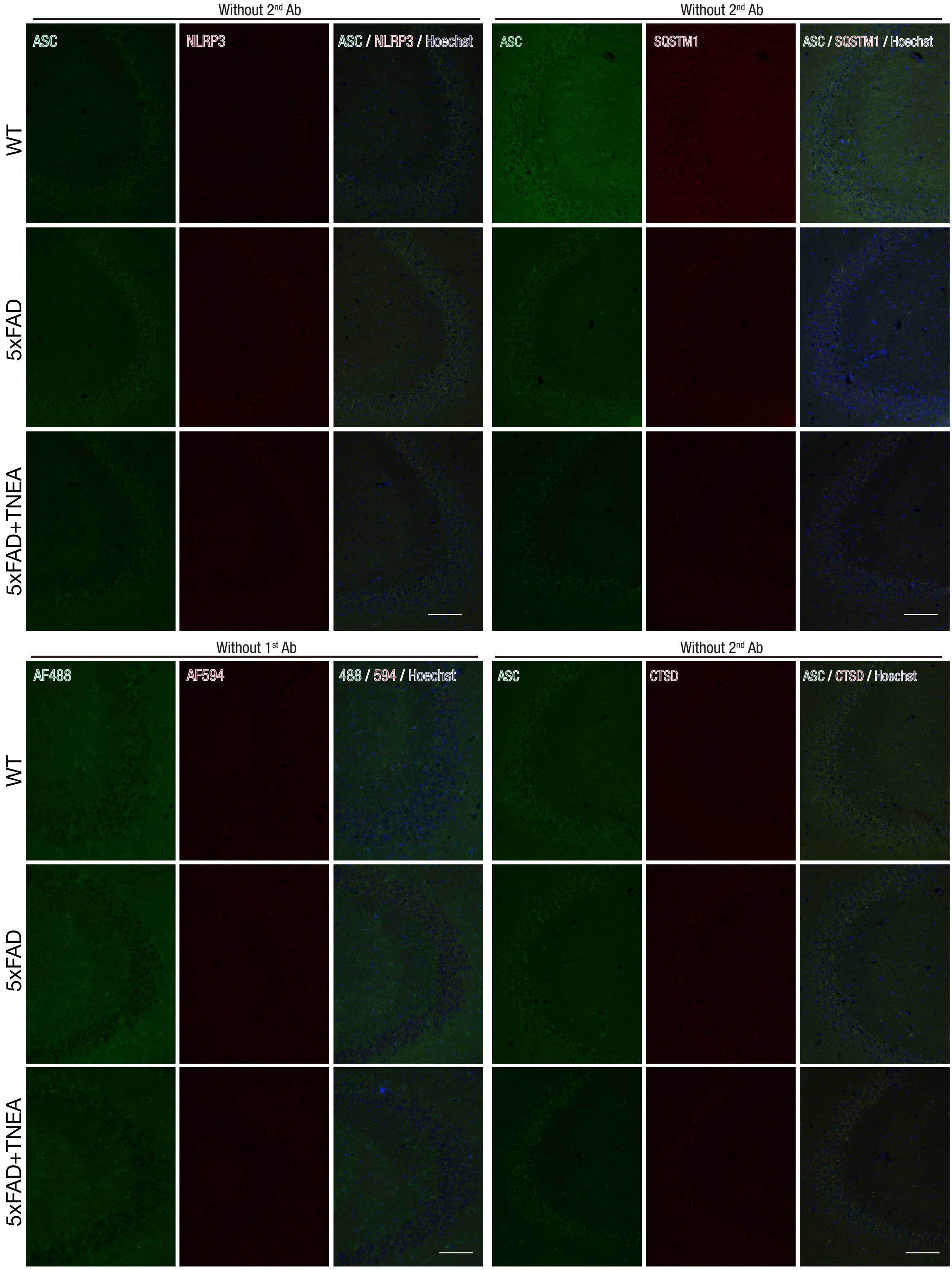

Fig. S3. Negative controls for the IHC of ASC, NLRP3, SQSTM1 and CTSD. The slides from each group were stained with each 1st antibody at the same dilution as performed in Fig.3 and 4, but without 2nd antibodies; or stained with 2nd antibodies at the same dilution as performed in Fig.3 and 4, but without 1st antibodies. Images were visualized at the same settings as performed in Fig.3 and 4. Scale bar: 100 μm.

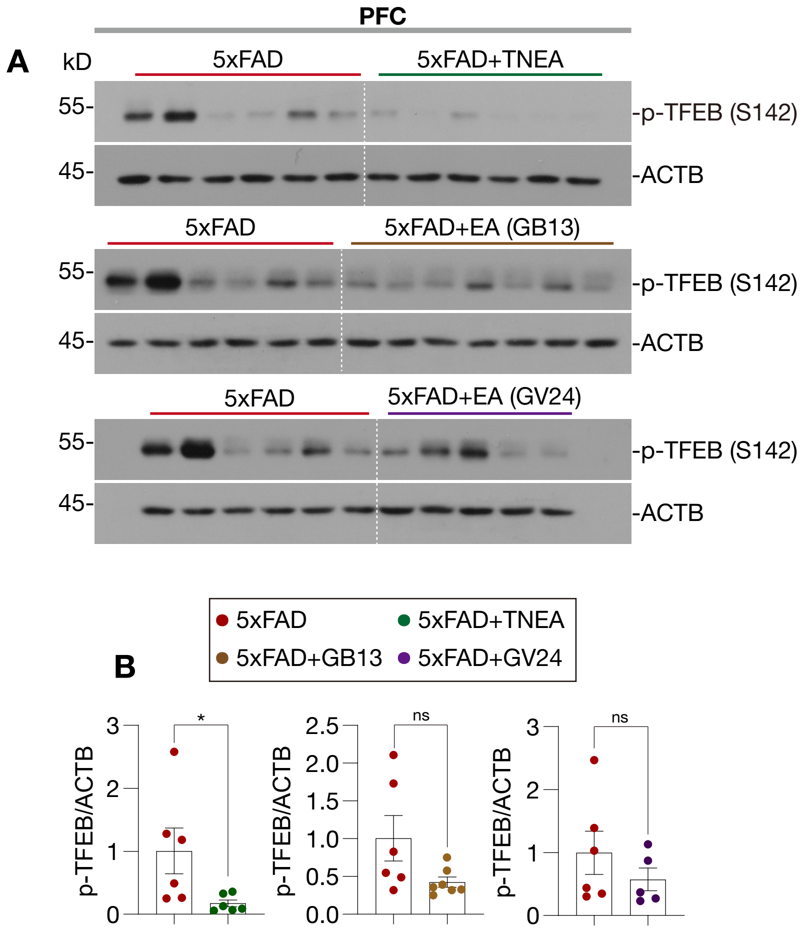

Fig. S4. Effects of TNEA and its composing acupoints on TFEB activation in the prefrontal cortex of 5XFAD mice. (**A**) Representative Western blots showed the levels of phosphorylated (*p*-) TFEB (S142) in the prefrontal cortex (PFC) of mice from each group. (**B**) Data are quantified as mean ± SEM (male, n = 5-7). **p<0.01, ns (p>0.05) vs. 5xFAD group analyzed by unpaired t-test, unpaired t-test with Welch's correction, or Mann Whitney U test

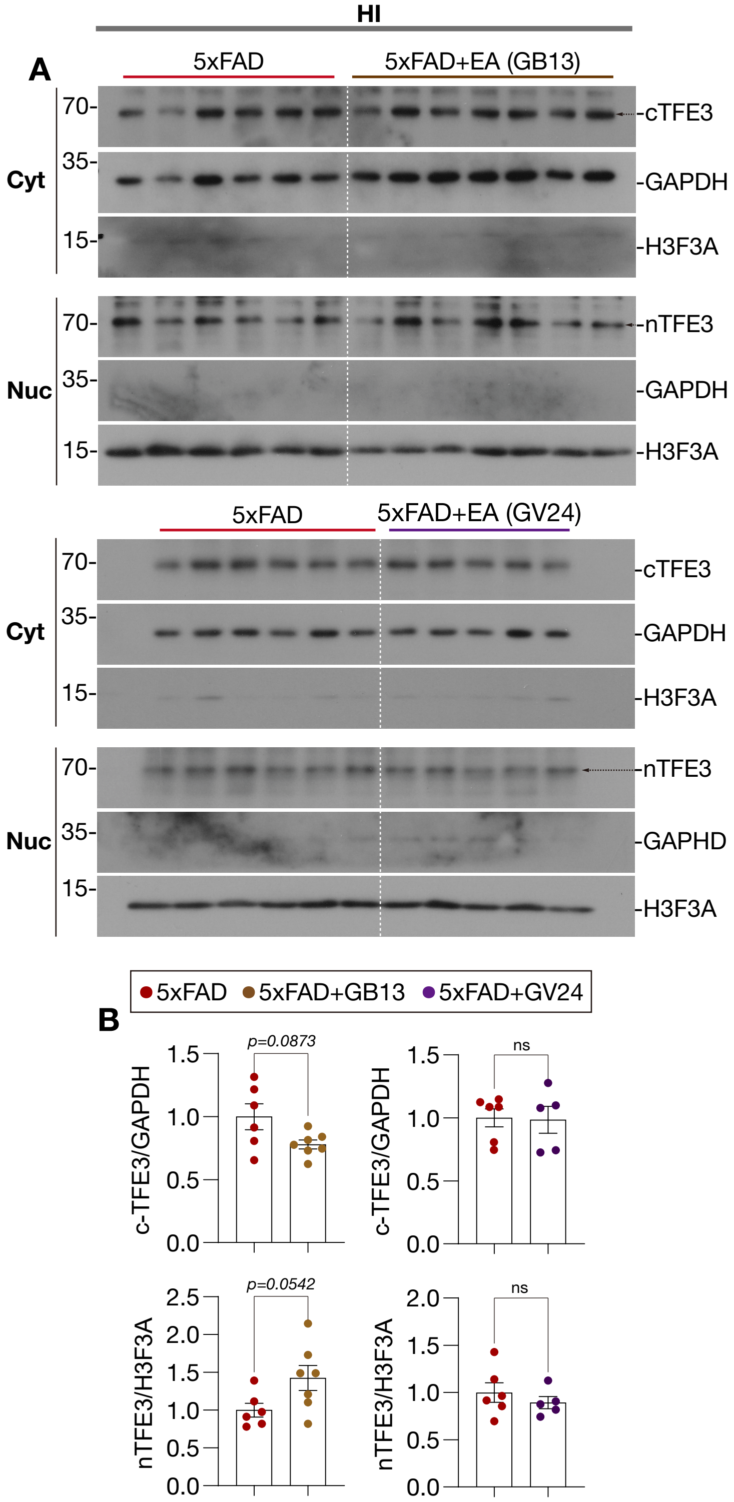

Fig. S5. Effects of the composing acupoints of TNEA on TFE3 activation in the hippocampi of 5xFAD mice. (**A**) Representative Western blots showed the levels of cytosolic (Cyt) /nuclear (Nuc) levels of TFE3 in the hippocampi (HI) of mice from each group. GAPDH and H3F3A (H3 histone) were used as cytosolic and nuclear loading controls respectively. (**B**) Data were quantified as mean ± SEM (male, *n*=5-7). **p*<0.05, ***p*<0.01, ns (*p*>0.05) vs. 5xFAD group analyzed by unpaired t-test.

Fig.1

C

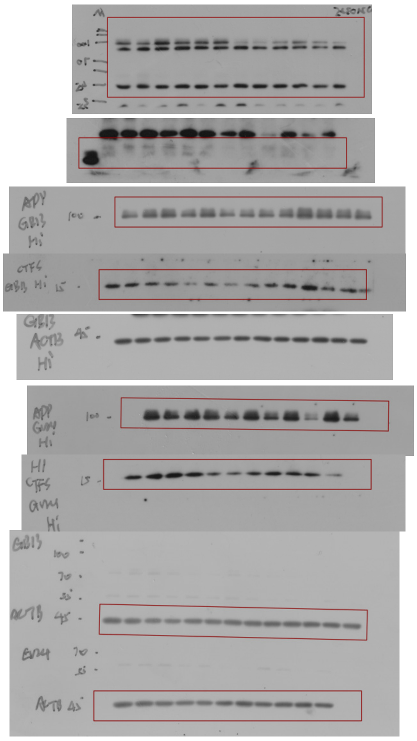

E

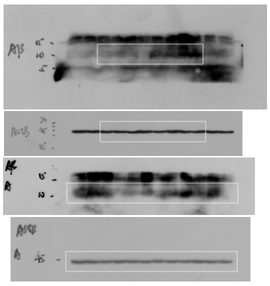

Fig.2

A

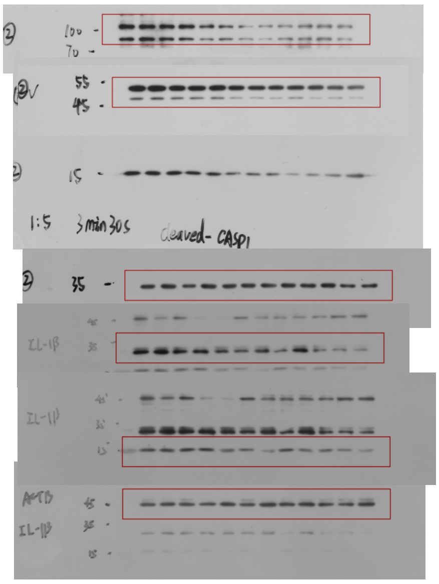

C

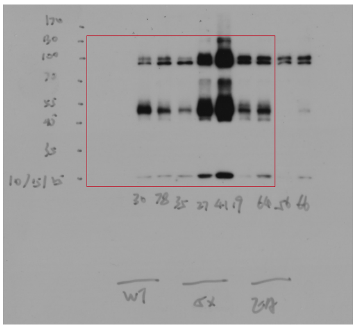

Fig.2

E

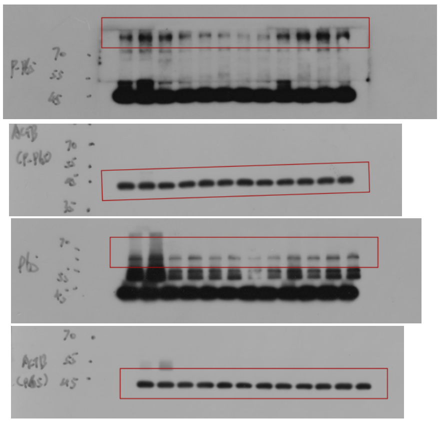

Fig.2

G

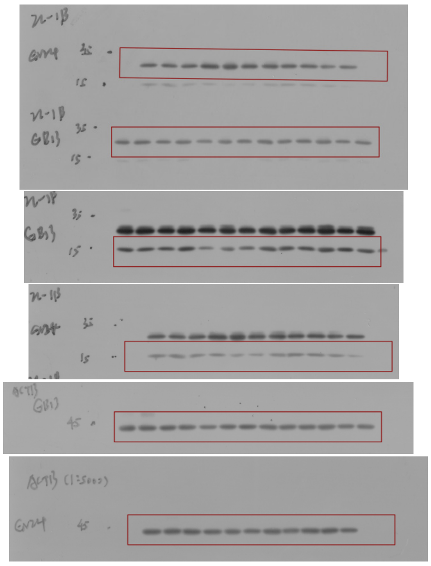

Fig. S6. Unprocessed scans of all immunoblots-1.

Fig.5

A

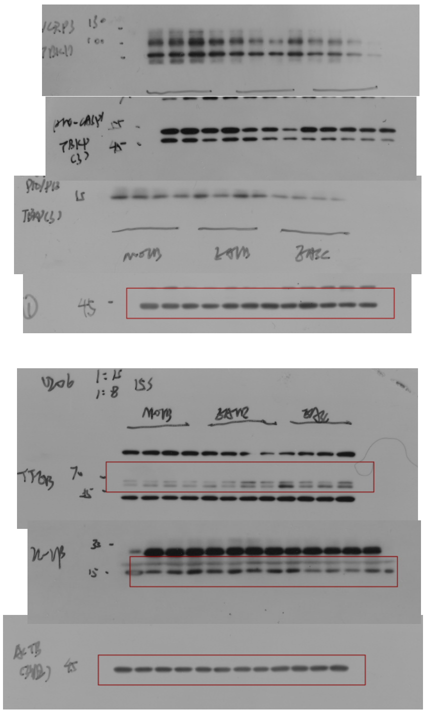

Fig.6

A

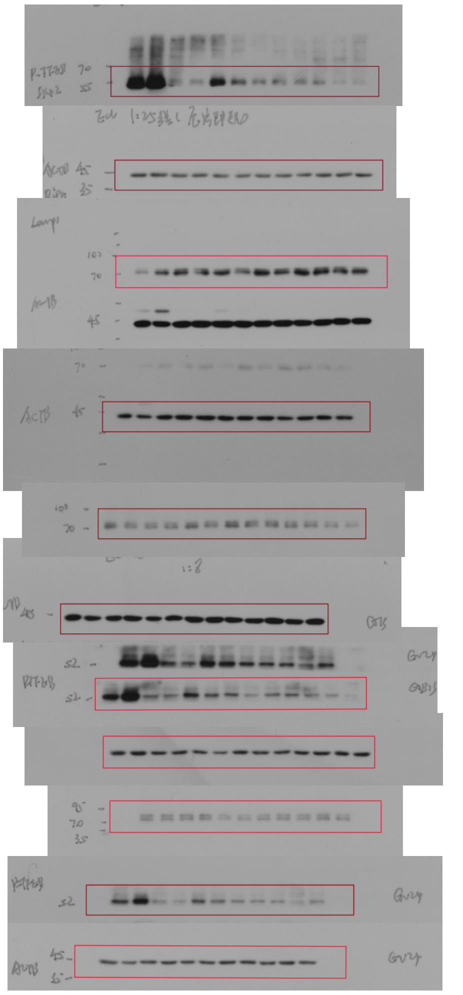

Fig.7

A

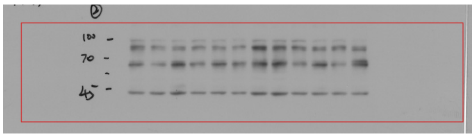

B

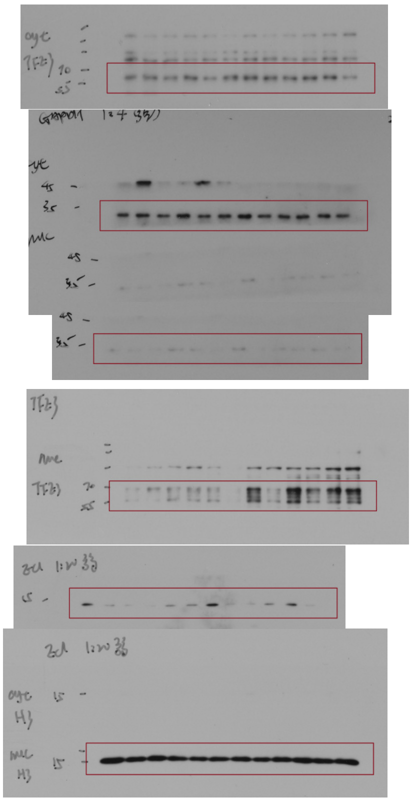

Fig.8

A

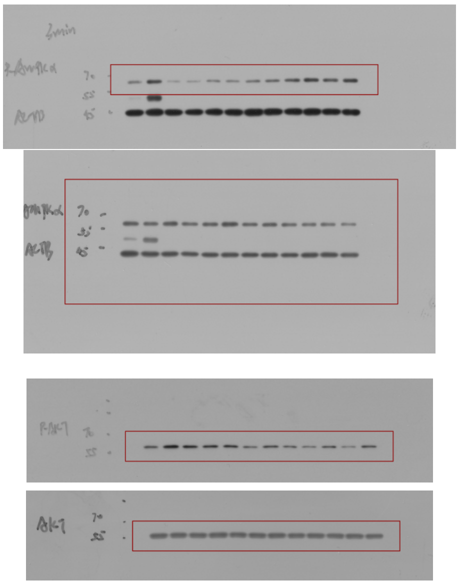

Fig. S6. Unprocessed scans of all immunoblots-2.

Fig.8

B

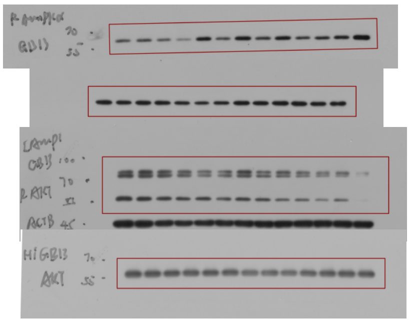

C

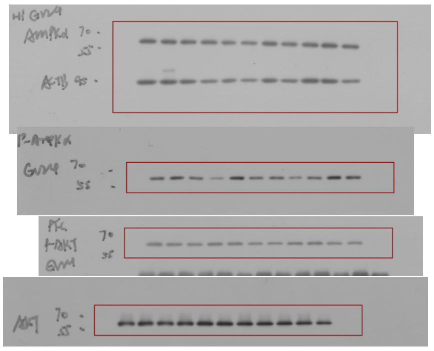

Fig.S1

A

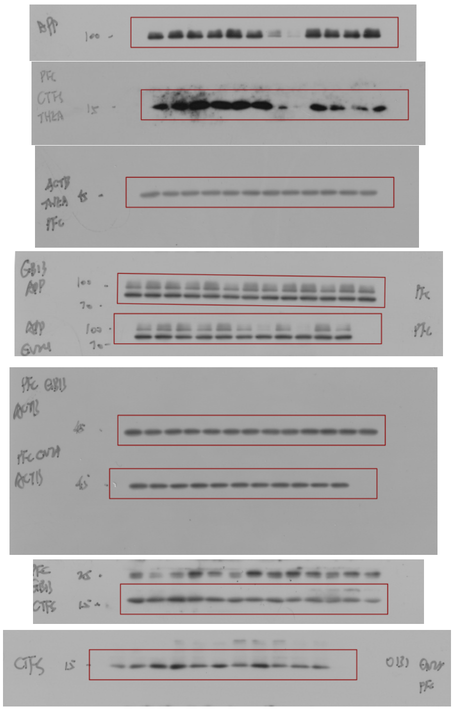

C

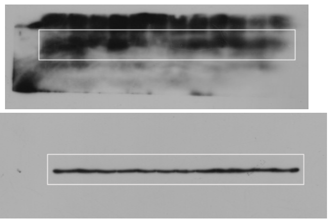

Fig.S2

A

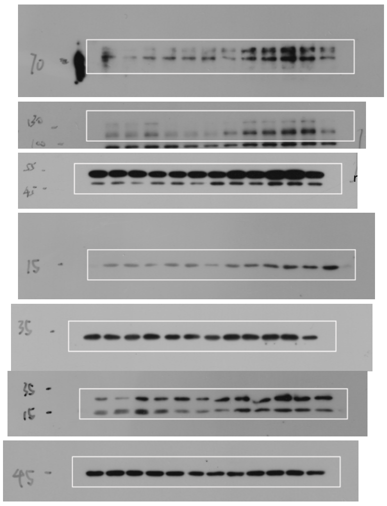

Fig.S2

C

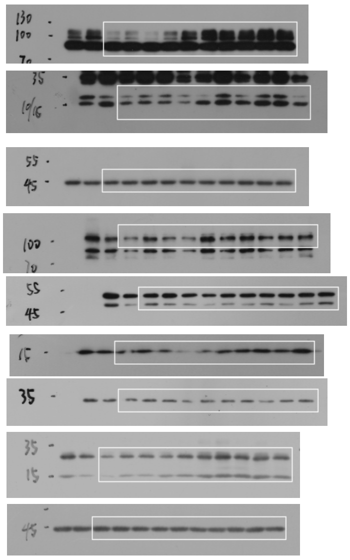

Fig. S6. Unprocessed scans of all immunoblots-3.

Fig.S3

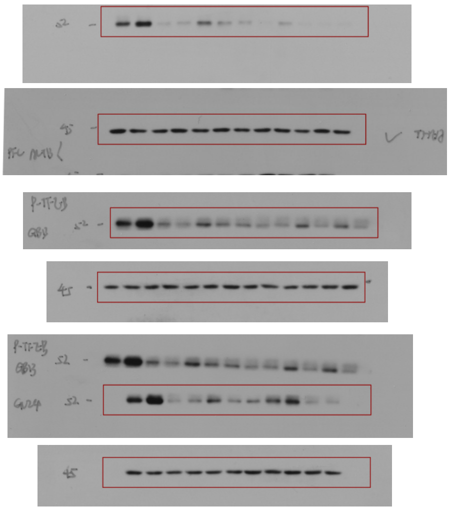

Fig.S4-GB13

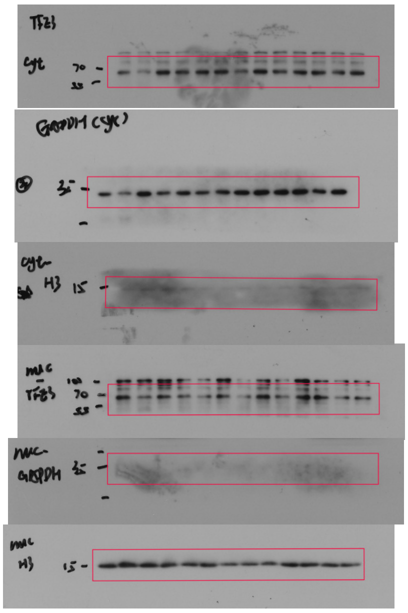

Fig.S4-GV24

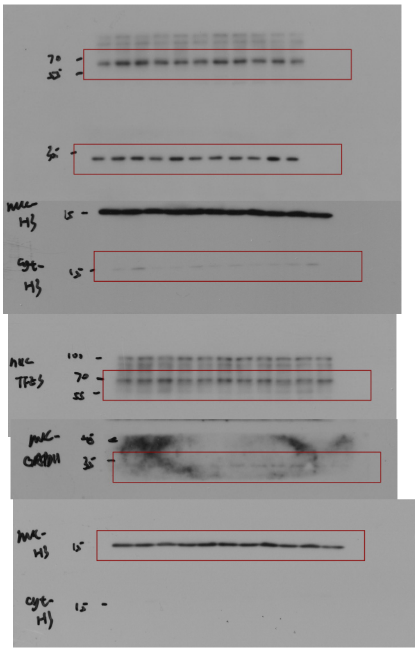

Fig. S6. Unprocessed scans of all immunoblots-4.
